# PCR-Based Multiple Species Cell Counting for *In Vitro* Mixed Culture

**DOI:** 10.1371/journal.pone.0126628

**Published:** 2015-05-13

**Authors:** Ruijie Huang, Junjie Zhang, X. Frank Yang, Richard L. Gregory

**Affiliations:** 1 Department of Pediatric Dentistry, West China Hospital of Stomatology, Sichuan University, Chengdu, Sichuan, China; 2 Department of Oral Biology, School of Dentistry, Indiana University, Indianapolis, Indiana, United States of America; 3 State Key Laboratory of Oral Diseases, West China Hospital of Stomatology, Sichuan University, Chengdu, Sichuan, China; 4 Department of Microbiology and Immunology, School of Medicine, Indiana University, Indianapolis, Indiana, United States of America; 5 Department of Pathology and Laboratory Medicine, School of Medicine, Indiana University, Indianapolis, Indiana, United States of America; Wageningen University, NETHERLANDS

## Abstract

Changes of bacterial profiles in microbial communities are strongly associated with human health. There is an increasing need for multiple species research *in vitro*. To avoid high cost or measurement of a limited number of species, PCR-based multiple species cell counting (PCR-MSCC) has been conceived. Species-specific sequence is defined as a unique sequence of one species in a multiple species mixed culture. This sequence is identified by comparing a random 1000 bp genomic sequence of one species with the whole genome sequences of the other species in the same artificial mixed culture. If absent in the other genomes, it is the species-specific sequence. Species-specific primers were designed based on the species-specific sequences. In the present study, ten different oral bacterial species were mixed and grown in Brain Heart Infusion Yeast Extract with 1% sucrose for 24 hours. Biofilm was harvested and processed for DNA extraction and q-PCR amplification with the species-specific primers. By comparing the q-PCR data of each species in the unknown culture with reference cultures, in which the cell number of each species was determined by colony forming units on agar plate, the cell number of that strain in the unknown mixed culture was calculated. This technique is reliable to count microorganism numbers that are less than 100,000 fold different from other species within the same culture. Theoretically, it can be used in detecting a species in a mixed culture of over 200 species. Currently PCR-MSCC is one of the most economic methods for quantifying single species cell numbers, especially for the low abundant species, in a multiple artificial mixed culture *in vitro*.

## Introduction

There are over 700 microbial species that live together in the oral cavity. They facilitate or compete with each other dynamically [1,2]. Many studies of oral bacterial interactions have been conducted in recent years [3–5]. The primary indicator typically used in multi-species study is the ratio or percentage of each species [2,6,7]. For *in vivo* multiple-species study, 16S rRNA clone analysis and recently mass amplicon sequencing are the major methods used to count the microbial cell number of each species within a mixed culture. For *in vitro* multiple species study, many current techniques can be used, such as fluorescence, antibiotic-resistance gene labeling, or mass amplicon sequencing. But fluorescence labeling costs time and is limited to less than five different species [8], antibiotic-resistance gene labeling is time-consuming [9], and mass amplicon sequencing is expensive for quantifying several species cell numbers in a lab with an average supply budget. None of the current methods stated above meets the criteria to be both economical and be applicable to more than 10 species, and those disadvantages largely limit multiple species research *in vitro*. The purpose of the present study was to introduce a new PCR-based multiple species cell counting (PCR-MSCC) technique that meets these two criteria.

## Materials and Methods

### Bacterial Strains and Growth Media

Ten microbial species were used: *Streptococcus mutans* (ATCC 700610; UA159), *Streptococcus gordonii* (ATCC 35105), *Streptococcus mitis* (ATCC 49456), *Streptococcus oralis* (ATCC 35037), *Streptococcus salivarius* (ATCC 27975), *Streptococcus sanguinis* (SK36, gift from Dr. Todd O. Kitten, Virginia Commonwealth University Philips Institute), *Staphylococcus aureus* (COL, gift from Dr. Steven R. Gill and Ann Gill, University of Rochester Medical Center), *Enterococcus faecalis* (ATCC 29212), *Lactobacillus casei* (ATCC 393) and *Staphylococcus epidermidis* (PR62A, gift from Dr. Steven R. Gill and Ann Gill). The ten species were selected based on the most abundant species reported by dental microbiome data [2], excluding anaerobic bacteria or bacteria without genome sequence information and including some other widely investigated pathogenic bacteria. *S*. *epidermidis* has been reported in endodontic lesions [10] and it is the fifth most abundant species observed in used tooth brushes [11]. *S*. *aureus* is also present in the oral cavity, but its numbers are negatively associated with *S*. *epidermidis* counts [12]. Unless otherwise stated, individual bacterial cultures were initiated and grown in Brain Heart Infusion plus 0.5% yeast extract (BHI+YE) broth, and the ten mixed species biofilm culture was grown in BHI+YE with 1% sucrose (BHI+YES). The incubation atmosphere was 5% CO_2_ at 37°C.

### Primer Design

To differentiate the ten species within a mixed culture and to count the cell number of each species, species-specific primers were designed. The primers used bacterial non-repeated chromosome sections as the template. Since each bacterial cell has only one chromosome, the DNA template copy number represents the bacterial cell number (if cell division and DNA synthesis are not considered). The species-specific sequence is a unique sequence of one species in a multiple species mixed culture. Species-specific primers were designed based on the species-specific sequences. Therefore, the species-specific primers would only amplify the species-specific sequence.

The BLAST tool on the NCBI website (http://blast.ncbi.nlm.nih.gov/Blast.cgi) was used to compare genomes and to find the DNA sequence for species-specific primer design. A random 10,000 bp chromosome sequence of one strain was compared with the genome of a second strain. From the Graphic Summary of the BLAST result, a 1000 bp sequence of the first strain with 0% identity to the second strain was identified. This sequence was then compared with the genome of the rest of the eight strains. If no identity was found in any of the rest of the strains, this sequence was defined as the species-specific sequence. If identities were found in one of the other strains, we repeated the process from the beginning with a second sequence or a third one until this sequence was proved to be different from all of the other genomes. In the present study, the species-specific sequence of each species was blasted with all of the rest of the species (45 comparisons in total), therefore all of the primers were unique to a certain species within the 10 species mixed culture. The primer-BLAST tool on the NCBI website (http://www.ncbi.nlm.nih.gov/tools/primer-blast/) was used to design the species-specific primers based on the species-specific sequence.

Since four of the strains used did not have their genome information available on the NCBI website, the genomes of *S*. *mitis* B6, *S*. *oralis* Uo5, *S*. *salivarius* CCHSS3 and *E*. *faecalis* V583 were used to surmise the genomes of *S*. *mitis* ATCC 49456, *S*. *oralis* ATCC 35037, *S*. *salivarius* ATCC 27975 and *Enterococcus faecalis* ATCC 29212, respectively.

### Primer Validation Test

To test the validation of primers that were designed based on surmised gene sequences and to further confirm the specificity of the primers used, polymerase chain reaction (PCR) was used to test the amplification product of each species whole DNA with all of the species-specific primers. Briefly, overnight bacterial cultures of each strain were individually grown in BHI+YE broth for 10 hours. Cells were harvested at their log phase and washed three times by PBS. Cells were processed as described before [13]. Buffer AL of DNeasy 96 Blood & Tissue Kit (QIAGEN, Valencia, CA) was added to each sample, which was then sonicated for 10 seconds (52% amplitude, Sonic Dismembrator, Model 500, Fisher Scientific) and repeated 5 times on ice. The sonicator tip was rinsed with 10% bleach followed by distilled water between different samples. The remaining DNA extraction steps were followed using the manufacturer's protocol. The quantity and quality of the extracted DNA were determined by NanoDrop 2000 (Thermo Fisher Scientific Inc., USA). The total DNA (100 ng) of each strain was amplified by all of the species-specific primers (0.25 μM) and TaKaRa Taq Polymerase (Chemicon International, Temecula, CA). The initialization step was 94°C 5 minutes, the amplification step had 30 cycles of 94°C 30 seconds, 55°C 30 seconds and 72°C 30 seconds, and the final elongation step was 72°C 10 minutes. The amplification product was confirmed by agarose gel electrophoresis.

### Validation Range Test of the Method

Each test has a validation range; if one result is beyond this range, it becomes invalid and should be excluded. Quantitative-PCR (q-PCR) was used to estimate the system error, which represents the maximum acceptable tolerance of cell number variations between samples in the present study. The DNA samples (200 ng) of each species were loaded in a MicroAmp Optical 96-well Reaction plate (Invitrogen, Grand Island, NY) with the primers (0.25 μM) of each species and Fast SYBR Green Master Mix (Applied Biosystems, Grand Island, NY). q-PCR amplification was performed on an ABI PRISM 7000 Sequence Detection System (Applied Biosystems). All of the default settings were used. The raw data (i.e., the data generated by *S*. *mutans* DNA versus all the primers from ten species) was extracted and normalized by the same species-primers paired data (i.e., the data generated by *S*. *mutans* DNA versus *S*. *mutans* species-specific primers). The amount of q-PCR product amplified by its own species-specific primers was defined as 1 (control), and the amount of products amplified by other primers were calculated based on their fold changes compared to control.

### q-PCR Efficiency Test

Since absolute PCR product quantification was used to estimate bacterial cell numbers, the q-PCR efficiency *E* of each paired strain-primers used in the present study was estimated. Briefly, the DNA sample of each strain was 1:2 serially diluted four times, and 2 μl of the undiluted and diluted samples were loaded with the species-specific primers for q-PCR as described before. Linear regression lines were used to estimate *E*. The x-value presented 1:2^n^ dilutions of DNA and the y-value presented Ct values. The slope (Δ*y* / Δ*x*), which represents *E*, was estimated by linear regression (Microsoft Office 2011, Version 14.1.0, Seattle, WA).

### Sample Preparation and q-PCR

There were two different mixed samples; one was the reference mixed sample and the other one was the unknown mixed sample. The former one would be used as the reference and the latter one would be the tested sample that we were interested in.

**Fig 1:**
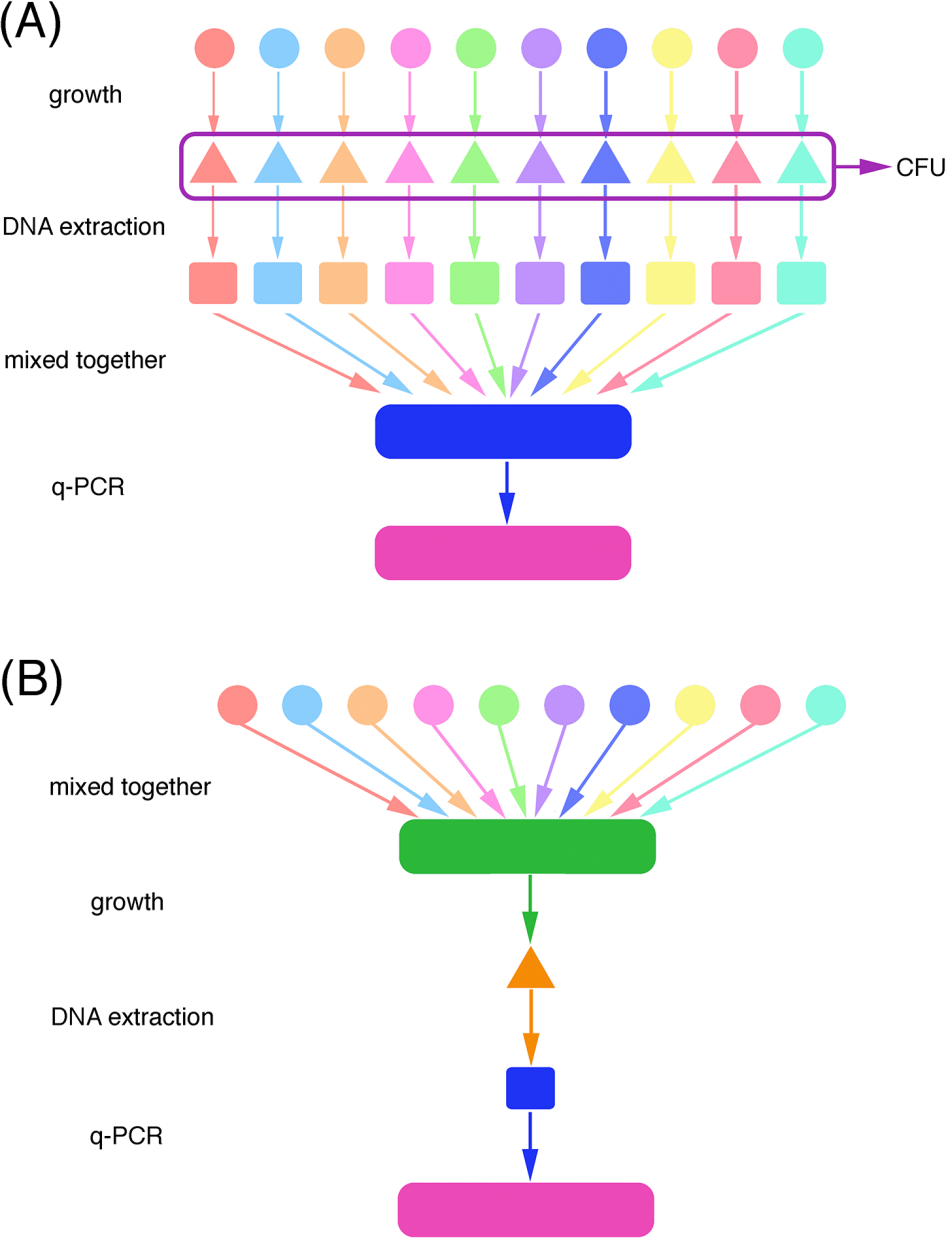
Schematic diagram of sample preparation. Panels A and B demonstrate the DNA processes of the reference mixed culture and the unknown mixed culture, respectively. Ten different colors in the first row represent 10 different species. Solid circles represent overnight bacterial cultures of each species, triangles represent the cultures that have grown for a stated period of time, and squares represent the DNA extracts from the samples.

To prepare the reference mixed sample, overnight cultures of the ten species were diluted 1:100 in BHI and inoculated individually for 10 hours (Fig 1A). After incubation, their individual colony forming units per ml (CFU/ml) were determined by spiral plating on blood agar plates. The DNA of those samples was extracted as described before, and the same amount (by volume) of samples from each DNA extraction were mixed. Since the ten species were mixed together, the original extracted DNA from each species was diluted 1:10.

To prepare the unknown mixed samples, according to microbiome data [2], overnight *S*. *mutans*, *S*. *gordonii*, *S*. *mitis*, *S*. *oralis*, *S*. *salivarius*, *S*. *sanguinis*, *S*. *aureus*, *E*. *faecalis*, *L*. *casei* and *S*. *epidermis* cultures were mixed in a 1:3:26:6:1:9:1:1:1:1 ratio (the percentages of *S*. *salivarius*, *S*. *aureus*, *E*. *faecalis*, *L*. *casei* and *S*. *epidermis* were not reported in Peterson et al., 2013, they were treated as 1 in the present study). The bacterial number of each strain in overnight broth was pre-calculated based on CFU/ml on agar plates, they were *S*. *mutans* 1.0×10^8^ CFU/ml, *S*. *gordonii* 5×10^7^ CFU/ml, *S*. *mitis* 4.0×10^8^ CFU/ml, *S*. *oralis* 1.3×10^8^ CFU/ml, *S*. *salivarius* 2.0×10^7^ CFU/ml, *S*. *sanguinis* 1.5×10^7^ CFU/ml, *S*. *aureus* 1.2×10^8^ CFU/ml, *E*. *faecalis* 1.1×10^8^ CFU/ml, *L*. *casei* 3.6×10^8^ CFU/ml and *S*. *epidermis* 4.0×10^8^ CFU/ml, respectively. To achieve the final 1:3:26:6:1:9:1:1:1:1 cell ratio, 61.5 μl of *S*. *mutans*, 369 μl of *S*. *gordonii*, 400 μl of *S*. *mitis*, 284 μl of *S*. *oralis*, 308 μl of *S*. *salivarius*, 3.69 ml of *S*. *sanguinis*, 51 μl of *S*. *aureus*, 56 μl of *E*. *faecalis*, 17.1 μl of *L*. *casei* and 15.4 μl of *S*. *epidermis* cultures were mixed together (Fig 1B). The mixed culture (1.0×10^6^ total CFU/ml, 5 ml/well) was grown in triplicate in BHI+YES for 24 hours in six-well-plates. Planktonic cells were discarded and the biofilm cells were harvested and washed three times by PBS. Half of the biofilm cells were discarded, by resuspending biofilm in 2 ml PBS and processing 1 ml of the suspension for DNA extraction, to limit the bacterial number within the DNA extraction kit capacity. Biofilm cell total DNA was extracted and processed for q-PCR as described before. The C_T_ value for the reference sample (control, *C*_*T*,*R*_) and the unknown sample (*C*_*T*,*X*_) of each species were recorded.

### Cell Quantification Algorithm

According to Livak and Schmittgen [14], the equation for the PCR exponential amplification is:

$$X_{n}=X_{0}\times {\left(1+E_{x}\right)}^{n}$$ (1)

where *X*_*n*_ is the molecule number amplified by q-PCR at cycle *n*, *X*_*0*_ is the initial molecule number, *E*_*x*_ is the amplification efficiency of the reaction, and *n* is the cycle number. For the reference (R) and unknown samples (X), they reached threshold at different threshold cycles (C_T_).

$$X_{T}=X_{0}\times {\left(1+E_{x}\right)}^{C_{T,X}}$$ (2)

$$R_{T}=R_{0}\times {\left(1+E_{R}\right)}^{C_{T,R}}$$ (3)

*C*_*T*,*R*_ and *C*_*T*,*X*_ are the threshold cycles for the reference and unknown samples, respectively. Since the reference and unknown samples belong to the same strain and the same primers are used, their thresholds and efficiencies are considered as the same. Thus,

$$X_{T}=R_{T}$$ (4)

$$E_{X}=E_{R}=E$$ (5)

dividing (2) by (3) and bringing Eqs (4) and (5) gives

$$\frac{X_{T}}{R_{T}}=\frac{X_{0}\times {\left(1+E_{x}\right)}^{C_{T,X}}}{R_{0}\times {\left(1+E_{R}\right)}^{C_{T,R}}}=\frac{X_{0}\times {\left(1+E\right)}^{C_{T,X}}}{R_{0}\times {\left(1+E\right)}^{C_{T,R}}}=1$$ (6)

rearranging provides the expression

$$\frac{X_{0}}{R_{0}}={\left(1+E\right)}^{C_{T,R}-C_{T,X}}$$ (7)

The reference microbial cell number (*N*_*R*_) processed for DNA extraction is estimated as

$$N_{R}=CFU_{R}\times V_{R}$$ (8)

where *CFU*_*R*_ is the cell concentration and *V*_*R*_ is the volume of processed cells. The percentage of sample lost during DNA extraction is assumed to be *k*. Moreover, in preparing the reference mixed culture of *q* different species, the DNA concentration of each species is diluted 1:q. Thus,

$$R_{0}=N_{R}(1-k)/q$$ (9)

and for the unknown mixed sample, without further dilution,

$$X_{0}=N_{X}(1-k)$$ (10)

where *N*_*x*_ is the specific microbial number in the unknown mixed culture.

Bringing (9) and (10) into (7) results in

$$\frac{N_{X}(1-k)}{N_{R}(1-k)/q}={\left(1+E\right)}^{C_{T,R}-C_{T,X}}$$ (11)

rearranging gives the expression

$$N_{X}={\left(1+E\right)}^{C_{T,R}-C_{T,X}}\times \frac{N_{R}}{q}$$ (12)

bringing (8) into (12) gives

$$N_{X}={\left(1+E\right)}^{C_{T,R}-C_{T,X}}\times \frac{CFU_{R}\times V_{R}}{q}$$ (13)

if the unknown mixed culture sample is diluted 1:t before DNA extraction to ensure appropriate cell numbers are processed for DNA extraction (≤2×10^9^ cells), the original bacterial number *N*_*X*,*O*_ is

$$N_{X,O}=N_{X}\times t$$ (14)

bringing (14) into (13) provides

$$N_{X,O}={\left(1+E\right)}^{C_{T,R}-C_{T,X}}\times CFU_{R}\times V_{R}\times \frac{t}{q}$$ (15)

This is the final equation to calculate the microorganism number in the mixed culture. If the reference single culture instead of the reference mixed culture is used and the unknown mixed culture sample is not diluted before DNA extraction, then q = 1 and t = 1, and the equation is

$$N_{X,O}={\left(1+E\right)}^{C_{T,R}-C_{T,X}}\times CFU_{R}\times V_{R}$$ (16)

## Results and Discussions

**Fig 2:**
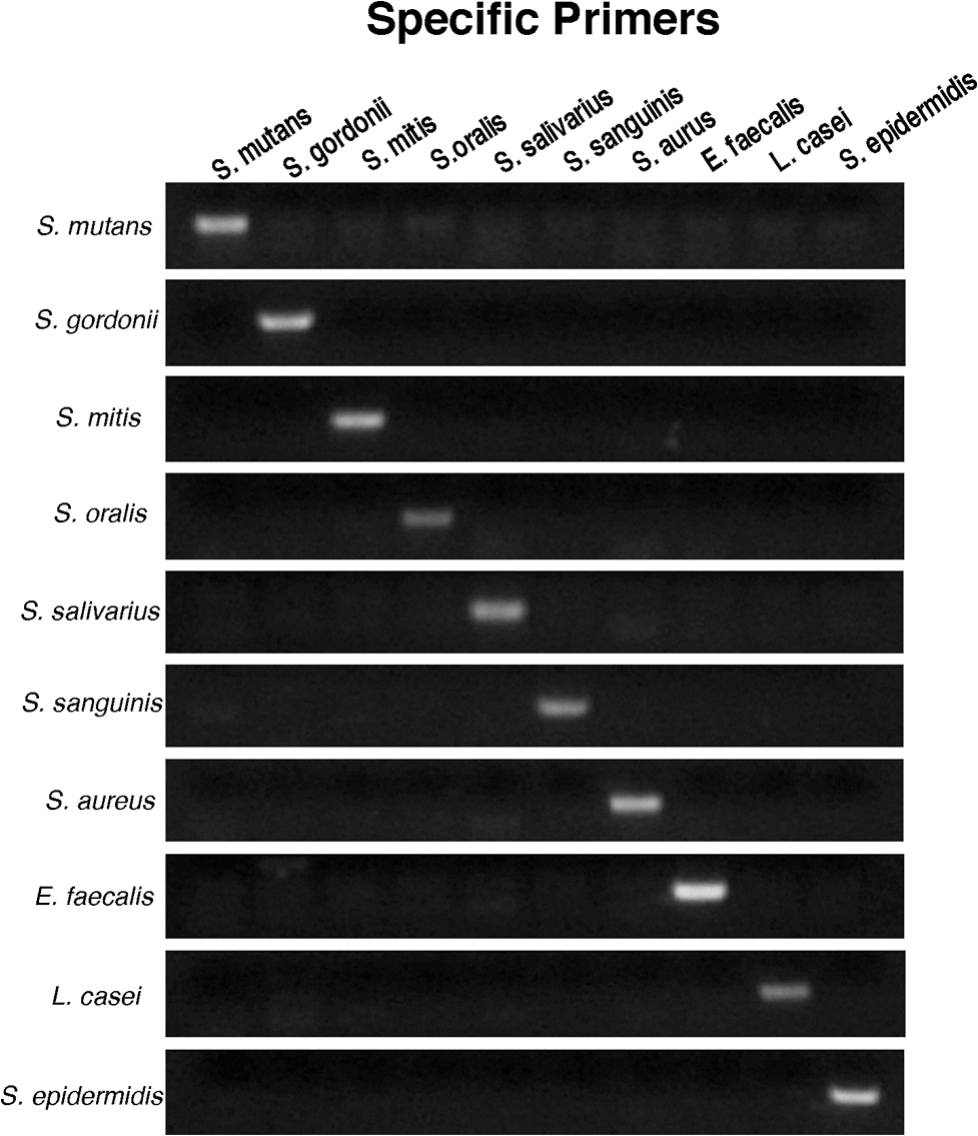
Primer validation. DNA of each species was loaded with the species-specific primers from each of the ten species for PCR. Each horizontal row represents the DNA extracted from one strain, and each vertical column represents the strain specific-primers of each strain.

The species-specific primers are listed in Table 1. The primer validation test results demonstrated that the DNA of each species was only amplified by its own species-specific primers (Fig 2), although the intensity of each band varied. Many factors affect band intensity of PCR products, such as Mg, Taq polymerase, DNA template and primer concentrations, primer length, GC content, primer/template ratio, thermocycler settings, etc. [15]. In addition, PCR product length, ethidium bromide concentration and shutter speed were noted to significantly affect band intensity. In the present study, GC content varied from 59.07 to 60.32% and PCR product length varied from 169 to 361 bp, while all of the other factors were the same for all samples. The DNA sample optical density absorbance at 260/280 nm was above 1.80. This primer specificity test may be skipped if the validation range test is used. Those two tests provide the same experimental results from semi-quantitative or quantitative aspects. The primer specificity test was included in the present study because it provided a direct visual confirmation that the primers were specific for each species. Its expense was less than the validation range test and was adequate for screening.

**Table 1:** Primer design.

	Sequence (5'->3')	Template strand
*S*. *mutans*	Forward primer	AGTCGTGTTGGTTCAACGGA
	Reverse primer	TAAACCGGGAGCTTGATCGG
*S*. *gordonii*	Forward primer	GCCTTAATAGCACCGCCACT
	Reverse primer	CCATCTCTGTTGTTAGGGCGT
*S*. *mitis*	Forward primer	CATCTCACGGGTTGAAGCCT
	Reverse primer	CCTCGCAGACTAAATTCGCC
*S*. *oralis*	Forward primer	GGCCGTGAGAATGTGATTGC
	Reverse primer	TGTTACAGCCTGACCACCAC
*S*. *salivarius*	Forward primer	CTGCTCTTGTGACAGCCCAT
	Reverse primer	ACGGGAAGCTGATCTTTCGTA
*S*. *sanguinis*	Forward primer	TCAGCAAATCCCCCAGGTTC
	Reverse primer	AACGGAGTGTCAGCGAAGTT
*S*. *aureus*	Forward primer	TCAGATGAGCAAGCTTCACCAA
	Reverse primer	TGGCTGTACTGCTGCTATACG
*E*. *faecalis*	Forward primer	CGCGAACATTTGATGTGGCT
	Reverse primer	GTTGATCCGTCCGCTTGGTA
*L*. *casei*	Forward primer	AAGAAAGGCTCACTGGTCGG
	Reverse primer	TTTTGGCCCGGATTCGATGA
*S*. *epidermidis*	Forward primer	CATATGGACCTGCACCCCAA
	Reverse primer	GCAACTGCTCAACCGAGAAC

The validation range test results indicated most of the errors occured at the 10^–6^ range, which means for PCR-MSCC any two species cell numbers should vary no more than 10^5^ if a 10% error is acceptable or no more than 10^4^ if a 1% error is acceptable (Table 2). Specifically, 4.4% errors occured at the 10^–4^ range, 25.6% errors occured at the 10^–5^ range, 56.7% errors occured at the 10^–6^ range and 13.3% errors occured at the 10^–7^ range. The system error may further be minimized by optimizing the primer design, such as changing primer length, altering terminal nucleotide, selecting a reasonable GC content and Tm, etc. [16]. The sensitivity and specificity of PCR-MSCC primer sets are dependent on the relative bacterial number in the mixed culture. If the cell number of each species is equal, the sensitivity and specificity are above 99.999%. But if one species is 10,000 less than all other species (suppose the number of other species are equal), the sensitivity and specificity become 91% and 99.9%, respectively. The larger the fold difference is, the lower the sensitivity and specificity are. If more species in the mixed culture results in a larger fold difference, it reduces the sensitivity and specificity.

**Table 2:** Validation Range Test of the Method.

		Primers									
		*S*. *mutans*	*S*. *gordonii*	*S*. *mitis*	*S*. *oralis*	*S*. *salivarius*	*S*. *sanguinis*	*S*. *aureus*	*E*. *faecalis*	*L*. *casei*	*S*. *epidermidis*
DNA	*S*. *mutans*	1	3.01×10^–6^	1.71×10^–6^	1.03×10^–6^	3.51×10^–6^	6.25×10^–7^	3.53×10^–6^	4.57×10^–6^	7.03×10^–7^	1.97×10^–6^
	*S*. *gordonii*	1.45×10^–5^	1	1.44×10^–5^	2.03×10^–6^	6.78×10^–6^	1.15×10^–6^	4.47×10^–6^	2.73×10^–5^	2.35×10^–5^	4.86×10^–6^
	*S*. *mitis*	2.92×10^–4^	1.67×10^–5^	1	2.38×10^–6^	3.92×10^–5^	4.57×10^–6^	8.05×10^–5^	1.68×10^–4^	1.58×10^–5^	8.06×10^–6^
	*S*. *oralis*	2.75×10^–5^	7.12×10^–6^	1.93×10^–5^	1	1.69×10^–5^	2.83×10^–5^	4.32×10^–5^	7.94×10^–5^	2.01×10^–5^	1.27×10^–5^
	*S*. *salivarius*	9.14×10^–6^	8.76×10^–6^	1.95×10^–6^	5.18×10^–7^	1	6.42×10^–7^	2.79×10^–6^	3.79×10^–6^	1.22×10^–6^	4.72×10^–5^
	*S*. *sanguinis*	4.03×10^–5^	3.53×10^–4^	2.91×10^–6^	1.37×10^–6^	2.28×10^–5^	1	2.16×10^–6^	9.07×10^–6^	1.65×10^–5^	1.92×10^–6^
	*S*. *aureus*	2.27×10^–6^	3.39×10^–5^	2.73×10^–5^	1.10×10^–6^	4.51×10^–6^	1.02×10^–6^	1	2.43×10^–6^	3.66×10^–6^	2.12×10^–6^
	*E*. *faecalis*	3.66×10^–6^	1.98×10^–4^	2.53×10^–6^	3.00×10^–7^	3.87×10^–6^	3.02×10^–7^	2.62×10^–6^	1	7.53×10^–7^	4.51×10^–6^
	*L*. *casei*	7.90×10^–6^	4.29×10^–6^	1.25×10^–5^	2.59×10^–7^	1.04×10^–6^	2.61×10^–7^	7.69×10^–7^	7.28×10^–7^	1	2.81×10^–6^
	*S*. *epidermidis*	7.63×10^–6^	6.33×10^–6^	3.30×10^–6^	8.19×10^–7^	4.32×10^–6^	2.59×10^–6^	9.39×10^–6^	7.74×10^–6^	2.27×10^–6^	1

DNA of each species was loaded with the species-specific primers from the ten species for q-PCR. The amount of q-PCR product amplified by its own species-specific primers was defined as 1 (control), and the amount of products amplified by other primers were calculated based on their fold changes compared to control. Each horizontal row represents DNA extracted from one strain, and each vertical column represents the species-specific primers of each strain.

**Fig 3:**
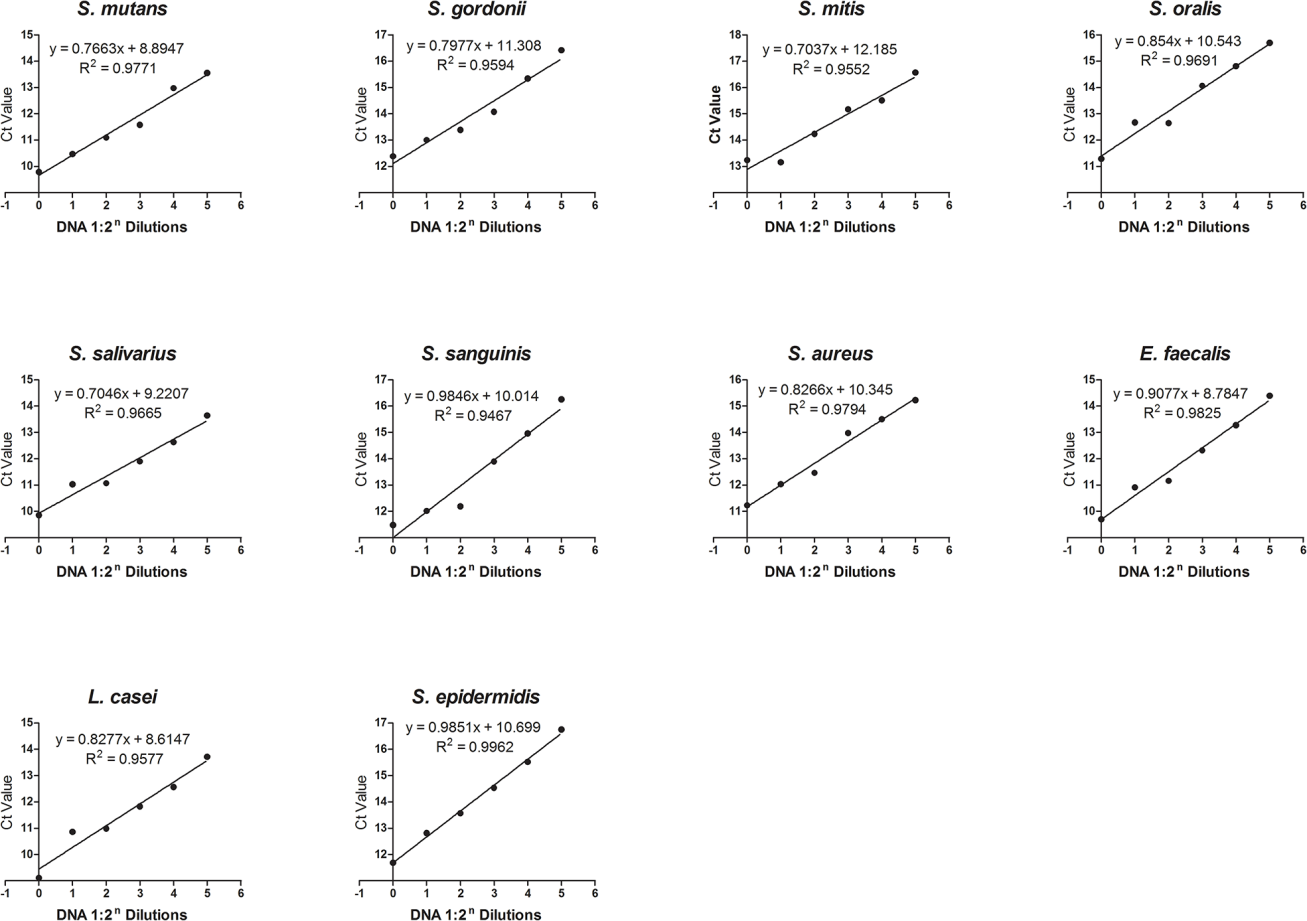
q-PCR efficiency test. The x-axis represents 1:2^n^ dilutions of DNA and the y-axis represents Ct values. Ideally, if one sample is diluted 1:2 (Δ*x* = 1), it will take one more cycle (Δ*y* = 1) to reach the same threshold. Ideally, the slope (Δ*y* / Δ*x*), which represents the amplification efficiency *E*, is equal to 1. For actual samples the slope is less than 1.

The q-PCR efficiency test implied the median *E* is 82.7% with the range from 70.0% to 98.5% (Table 3). The coefficient of determination (R^2^) of every regression line was equal to or larger than 0.95 (Fig 3). For most of the q-PCR efficiency studies, DNA template concentrations (log_10_X) were used as the X-axis, and the efficiency calculation equation was *E* = 10^−1/*slope*^−1 with the ideal slope of -3.32 [17–19]. In the present study, we changed the X-axis to 1:2^n^ dilutions of DNA template, this made the efficiency much easier to predict because *E = slope*. However, if the absolute DNA template will be used, the X-axis could be stated as (log_2_ *X*)^−1^. R^2^ in the present study could approach 1 by adding five more DNA template dilution points for the linear regression [18,19]. The amplification condition of each species was not optimized because in optimizing there would be 10 separate PCR amplifications, one for each species. It is practical with 10 species, but may not be practical with more than 20 species. In a long run point of view, the default PCR set-up instead of an optimized condition for each species was used.

**Table 3:** Microbial cell quantification.

	*E*	*C*_*T*,*R*_	*C*_*T*,X_*	*CFU*_*R*_	*V*_*R*_	*t*	*q*	*N*_*X*,*O*_
*S*. *mutans*	0.7663	13.00	16.49	3.28×10^8^	5	2	10	**4.51×10** ^**7**^
*S*. *gordonii*	0.7977	14.34	15.24	4.79×10^7^	5	2	10	**2.82×10** ^**7**^
*S*. *mitis*	0.7037	15.86	19.95	5.41×10^8^	5	2	10	**6.11×10** ^**7**^
*S*. *oralis*	0.8540	14.75	13.80	4.40×10^8^	5	2	10	**7.92×10** ^**8**^
*S*. *salivarius*	0.7046	12.79	11.46	1.41×10^8^	5	2	10	**2.87×10** ^**8**^
*S*. *sanguinis*	0.9846	13.55	13.07	2.14×10^7^	5	2	10	**2.98×10** ^**7**^
*S*. *aureus*	0.8266	13.38	20.28	2.63×10^8^	5	2	10	**4.13×10** ^**7**^
*E*. *faecalis*	0.9077	11.51	13.15	4.48×10^8^	5	2	10	**1.56×10** ^**8**^
*L*. *casei*	0.8277	12.19	16.80	1.29×10^8^	5	2	10	**8.00×10** ^**7**^
*S*. *epidermidis*	0.9862	14.84	20.30	1.91×10^7^	5	2	10	**4.48×10** ^**7**^

The cell number of each species within the mixed species biofilm (*N*_*X*,*O*_) was calculated by equation [15]. *E* is the amplification efficiency of the reaction, *C*_*T*,*R*_ and *C*_*T*,*X*_ are the threshold cycles (C_T_) for the reference mixed and unknown mixed cultures, respectively. *CFU*_*R*_ are the colony forming units for the reference bacterial cell cultures. *V*_*R*_ are the volumes of standard bacterial cell cultures processed for DNA extraction, *t* is the fold dilution of the unknown mixed cultures, and *q* is the number of multiple species whose DNA extractions were equally mixed together to serve as the reference mixed culture. The asterisk(*) indicates the mean value of triplicate samples.

The cell quantification results demonstrated the cell number of the ten species varied from 4.48×10^5^ to 7.92×10^8^ cells. Since the most significant difference was at the 10^3^ range, all of the values were considered valid (Table 3). The maximum difference occured between *S*. *oralis* (7.92×10^8^) and *S*. *epidermidis* (4.48×10^5^). The difference was 1.77×10^3^ fold. If the *S*. *oralis* number was multiplied by the system error of the *S*. *oralis* DNA template with *S*. *epidermidis* primers then (7.92×10^8^) × (1.27×10^–5^) = 6.23×10^3^ cells. This number is very small compared to the actual *S*. *epidermidis* number. If it was large, the raw *S*. *epidermidis* data should be adjusted by subtracting the augmented data generated by *S*. *oralis*.

The cell number of each species in the mixed cultures can be estimated through either regular PCR or q-PCR. The present study has demonstrated the q-PCR method. If the PCR method is used, the PCR band intensity should be semi-quantified by image software (i.e., ImageJ), and the final equation will be

$$N_{X,O}=\frac{I_{R}}{I_{X}}\times CFU_{R}\times V_{R}\times \frac{t}{q}$$ (17)

where *I*_*R*_ and *I*_*X*_ are the band intensities of the reference mixed and unknown mixed cultures, respectively.

The sequences targeted by the primers can be replicated within the target genome if the fast growing cell is dividing and synthesizing DNA. Suppose n% of our reference cells are undergoing replication when they are harvested. The reference cell number is A, and the actual sequence copy number is (1+n%)×A. Suppose the unknown cell number is B, then the actual unknown sample sequence copy number is (1+n%)×B. When divided (1+n%)×B by (1+n%)×A, the coefficient (1+n%) is gone. So this will not affect the final result.

There are several methods used for microbial quantification (Table 4). Fluorescence *in situ* hybridization (FISH) is the best option for microbial quantitation *in situ*. The average laboratory typically has access to a general confocal laser scanning microscope that can differentiate three to five different fluorescent wavelengths, but microscopes of ten different channels are rare and very expensive [8,20,21]. The cell quantification result of qPCR strongly correlated with the FISH results as reported by Ammann et al [22]. The antibiotic resistance gene labeling method costs time and very limited species could be analyzed at one time [5]. Colony morphology recognition on selective agar plates is available for nine species [23]. Microbiota sequencing is time efficient and works well with ten species, but the expense is relatively high, approximately $50-$200 per sample. The expense for PCR-MSCC is approximate $1–$2 per species. PCR-MSCC is superior to microbiota analysis for targeting less than 50 different species in the mixed culture, or for targeting a rare species (more than 10^3^ fold less than other species). However, on the other hand, microbiota analysis is superior to PCR-MSCC for targeting more than 50 different species and for *in vivo* samples with unknown species. For mass sequencing (454/Roche GS FLX) the mean error rate is 1% [4,24] and it could quantify cells of one species up to 10^3^ fold than other species [25], while for PCR-MSCC the mean error rate is 0.001% and it could quantify cells of one species up to 10^5^ fold than other species.

**Table 4:** Comparison of different cell quantification methods.

	Tolerance for fold difference between species	Expense	Workable species	Quantify live cell number	Bacterial strains	Sample status
**PCR-MSCC**	~10^5^	$1–2/species	~200	-	known	in vitro
**Mass sequencing**	~10^3^	$50–200/sample	~1000	-	unknown	in vitro, in vivo
**Selective agar**	~10^2^–10^3^	$1/species	~10	+	known	in vitro
**FISH** *	~10^2^–10^3^	$10–20/sample	~5	-/+	known	in vitro, in situ

*FISH: Fluorescent in situ hybridization.

Theoretically, PCR-MSCC can be applied to over 200 species because the primers can target any region of the genome, and are not limited to the 16S rRNA region. For two species of 93% identities, for instance, if the genome size of one is ~2,000,000 bp, there will be 2,000,000×(1–93%) = 140,000 bp region that can be used for species-specific primer design. If each DNA sequence cloned by the primers is 500 bp, the total available length is enough to detect 280 mixed species. Ammann et al reported a q-PCR quantification method for a ten mixed species subgingival biofilm model, in which they used 16S rRNA for template design [22]. The study of Ammann and co-workers indicated that for communities of limited complexity, also 16S rRNA targeted approaches can be used for quantitative analysis of microbial composition.

PCR-MSCC can also be used to compare different strains within the same species, such as *S*. *mutans* UA159 versus *S*. *mutans* GS-5. PCR-MSCC was defined as PCR-based multiple species cell counting. Actually, PCR-based multiple strain cell counting might be more appropriate because it is designed based on strain instead of species. Since in the present study, ten different species instead of strains were used, the name with species was used. PCR-MSCC is limited to *in vitro* studies to date. But it is still important because *in vitro* studies are the foundation of *in vivo* studies and most mechanistic explorations are conducted *in vitro*. PCR-MSCC can be used in *in vivo* studies only if the genome sequences of the majority species are known. Future studies will focus on analyzing the genome sequences of the oral microbiome and designing species-specific primers for the majority species or pathogenic species. In the far future, PCR-MSCC may be used in clinical diagnostics to monitor core microbiome profile changes.

One limitation with species-specific primer design is the limited bacterial genome information and the limited number of available bacterial strains. We had been trying to work with fully sequenced strains, but we failed because of at least one of the following reasons, no genome information was available for that species, the strain was available from ATCC but its sequence was unknown, or the sequence of the strain was known but it was not available from ATCC or could not be shipped to the US. But at least for the latter two situations, we could surmise the sequence and test it. To design primers based on a different strain of the same species is a gamble. In the present study, the species-specific primers of seven out of ten species were found in the first trial, two in the second trial, and the last one in the fifth trial. This problem should not be a permanent barrier because ATCC is frequently collecting newly sequenced strains and the genome information of many bacterial strains under sequencing will be posted in the future.

In conclusion, currently PCR-MSCC is one of the most economic methods for quantifying single species cell numbers, especially for the low abundant species, in a multiple artificial mixed culture *in vitro*.
